# Complete mitogenome and phylogenetic analysis of the tertiary relict shrub *Platycrater arguta* (Hydrangeaceae)

**DOI:** 10.1080/23802359.2026.2732717

**Published:** 2026-09-18

**Authors:** Ke Guo, Zhi-Cheng Zhu, Meng-Ru Liu, Xi Liu, Yong-Hua Zhang

**Affiliations:** ^a^ College of Life and Environmental Science, Wenzhou University, Wenzhou, China; ^b^ Qingyuan Conservation Center of Qianjiangyuan-Baishanzu National Park, Qingyuan, China; ^c^ Zhejiang Wuyanling National Nature Reserve Management Bureau, Wenzhou, China

**Keywords:** *Platycrater arguta*, mitogenome, Hydrangeaceae, phylogeny, trans-splicing events

## Abstract

*Platycrater arguta* Siebold & Zucc., is a monotypic Hydrangeaceae genus, yet its mitogenomic information remains limited. We assembled its mitogenome from PacBio HiFi reads: 435,757 bp (45.76% GC), containing 66 genes (36 protein-coding, 27 tRNA, 3 rRNA). Complex trans-splicing introns occurred in *nad1*, *nad2* and *nad5*; codons were strongly AT-biased. It also had 100 large dispersed repeats (>50 bp), 26 tandem repeats and 136 SSRs. Using shared PCGs from 21 species, P. arguta fell within Hydrangeaceae, sister to *Hydrangea macrophylla* and *H. chinensis* (100% bootstrap). This mitogenome is a valuable resource for Hydrangeaceae phylogeny and comparative genomics.

## 1. Introduction

*Platycrater arguta* Siebold & Zucc. (Hydrangeaceae), commonly known as “Zhuwang’e” in China due to its distinctive spider web-like bracts, is a rare and endangered tertiary relict shrub endemic to East Asia (Wei and Bartholomew 2001; Qiu et al. 2009). This monotypic genus is discontinuously distributed in montane vegetation of eastern China and southern Japan (Qiu et al. 2009; Zhang et al. 2025). As a paleo-endemic relic with significant phylogeographic and evolutionary value, *P. arguta* has been designated as a nationally protected plant in China (Qi et al. 2014; Zhang et al. 2025). Previous molecular phylogeographic studies of *P. arguta* have primarily focused on chloroplast DNA (cpDNA) and nuclear markers to elucidate the evolutionary history and population dynamics of this disjunctly distributed species (Qi et al. 2014; Y. Qiu et al. 2009). Additionally, research on *P. arguta* has addressed its reproductive ecology, physiological responses to abiotic stress, conservation strategies (Zhang and Qiu 2017; Xiahou et al. 2019; Wang et al. 2021). Here, we presented the complete mitogenome of *P. arguta*, assembled and annotated using PacBio long-read sequencing technologies. We comprehensively analyzed its structural features and reconfirmed its phylogenetic position.This study not only advances organelle genomics research in this relict species, but also provides a foundation for the development of conservation genetic technologies.

## 2. Materials and methods

**Figure 1.**
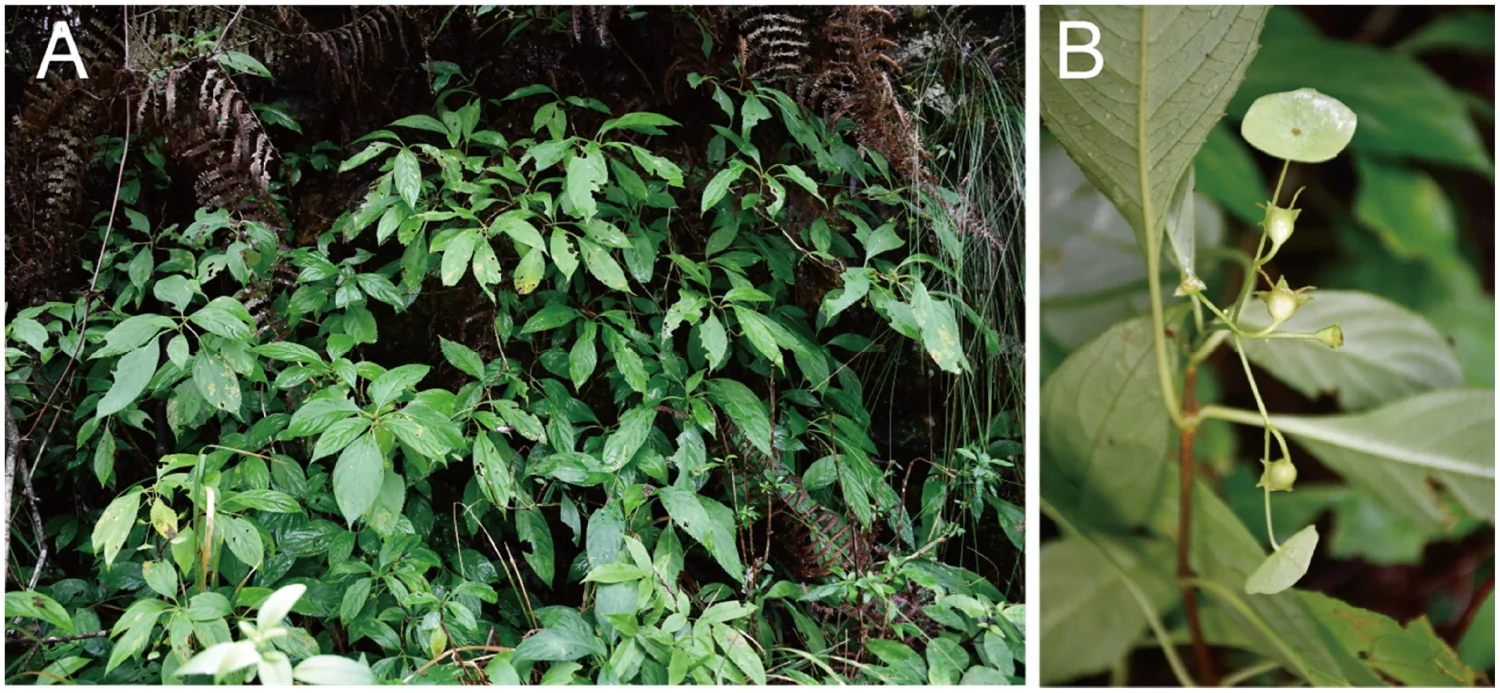
Morphological features of *P. arguta*. (A) The plant in its natural habitat. (B) Close-up of an infructescence, showing the persistent, accrescent calyx lobes and immature fruits. Photos by Yong-Hua Zhang.

Fresh leaves of *P. arguta* (Figure 1) were collected from Baipu Gou, Baishanzu Town, Qingyuan County, Lishui City, Zhejiang Province, China (27°45.08′N,119°11.56′E). A voucher specimen (ZYH230520) has been deposited at the Herbarium of Wenzhou University (WZU). Research material requests should be directed to Yong-Hua Zhang (zhangyhua@wzu.edu.cn). After collection, fresh tissue samples were immediately frozen in liquid nitrogen and stored at −80 °C to maintain DNA integrity. Total DNA was extracted using the DNeasy Plant Mini Kit (Qiagen,Valencia, CA, USA), then sequenced using PacBio HiFi mode on the Sequel II platform at Bena Genomics (Wuhan, China).

Based on the 10-Gb subset of the PacBio HiFi reads (with a read N50 of 18,092 bp), we performed de novo assembly of the mitogenome using PMAT2 v2.1.5 (Han et al. 2025) and OATK v1.0 (Zhou et al. 2024), with both strategies yielding largely consistent genomic structures. The OATK assembly was selected as the final reference. The initial complex graph was visualized using Bandage v0.8.1 (Wick et al. 2015). All HiFi reads were mapped to assembled contigs using minimap2 v2.24 (Li 2018), and based on the highest-supported connection evidence, contigs were ordered and oriented into a master circular molecule of 435,757 bp. For standardization, the sequence was linearized and rearranged at the start codon of the conserved *cox1* gene. The HiFi reads from this 10-Gb subset were aligned to the final sequence using minimap2, and average sequencing depth was calculated using SAMtools v1.16 (Danecek et al. 2021). The mitogenome was annotated using IPMGA (Li et al. 2025), with tRNA and rRNA genes verified using tRNAscan-SE v2.0.12 (Schattner et al. 2005) and BLAST+ v2.12.0 (Chen et al. 2015), respectively. Start/stop codons and intron-exon boundaries of protein-coding genes were manually curated. The complete mitogenome map was drawn using PMGmap (Zhang et al. 2024a,^b^).

All protein-coding genes (PCGs) were extracted using PhyloSuite v1.2.3 (Zhang et al. 2020), and their Relative Synonymous Codon Usage (RSCU) was analyzed using CodonW v1.4.2. Repetitive sequences were identified using multiple tools: REPuter (≥ 30 bp, Hamming distance 3) for dispersed repeats, Tandem Repeats Finder v4.09 for tandem repeats (match/mismatch/indel: 2, 7, 7), and MISA for SSRs (minimum repeats: 10, 5, 4, 3, 3, 3 for mono- to hexanucleotides). Mitochondrial plastid DNA (MTPTs) was identified by aligning the chloroplast genome against the mitogenome using BLASTn, with homologous fragments > 100 bp and > 90% identity considered MTPTs.

To determine the phylogenetic position of *P. arguta*, a Maximum Likelihood (ML) analysis was performed using mitochondrial protein-coding genes from 21 species, with *Arabidopsis thaliana* as the outgroup (accession numbers in Supplemental Table S1). All PCGs were extracted by PhyloSuite v1.2.3 by the ‘Concatenate Sequence’ tool, aligned using MAFFT v7.505 (Katoh and Standley 2013) under the ‘Codon’ alignment mode using the standard genetic code and the automatic strategy, and trimmed with Gblocks v0.91b (Castresana 2000) under the ‘Codons’ data type. The ML tree was inferred using IQ-TREE v2.2.0 (Minh et al. 2020), with best-fit models determined by ModelFinder (Kalyaanamoorthy et al. 2017) under the Bayesian Information Criterion (BIC). Branch support was assessed with 1,000 bootstrap replicates. The tree was visualized using FigTree v1.4.4.

## 3. Results

**Figure 2.**
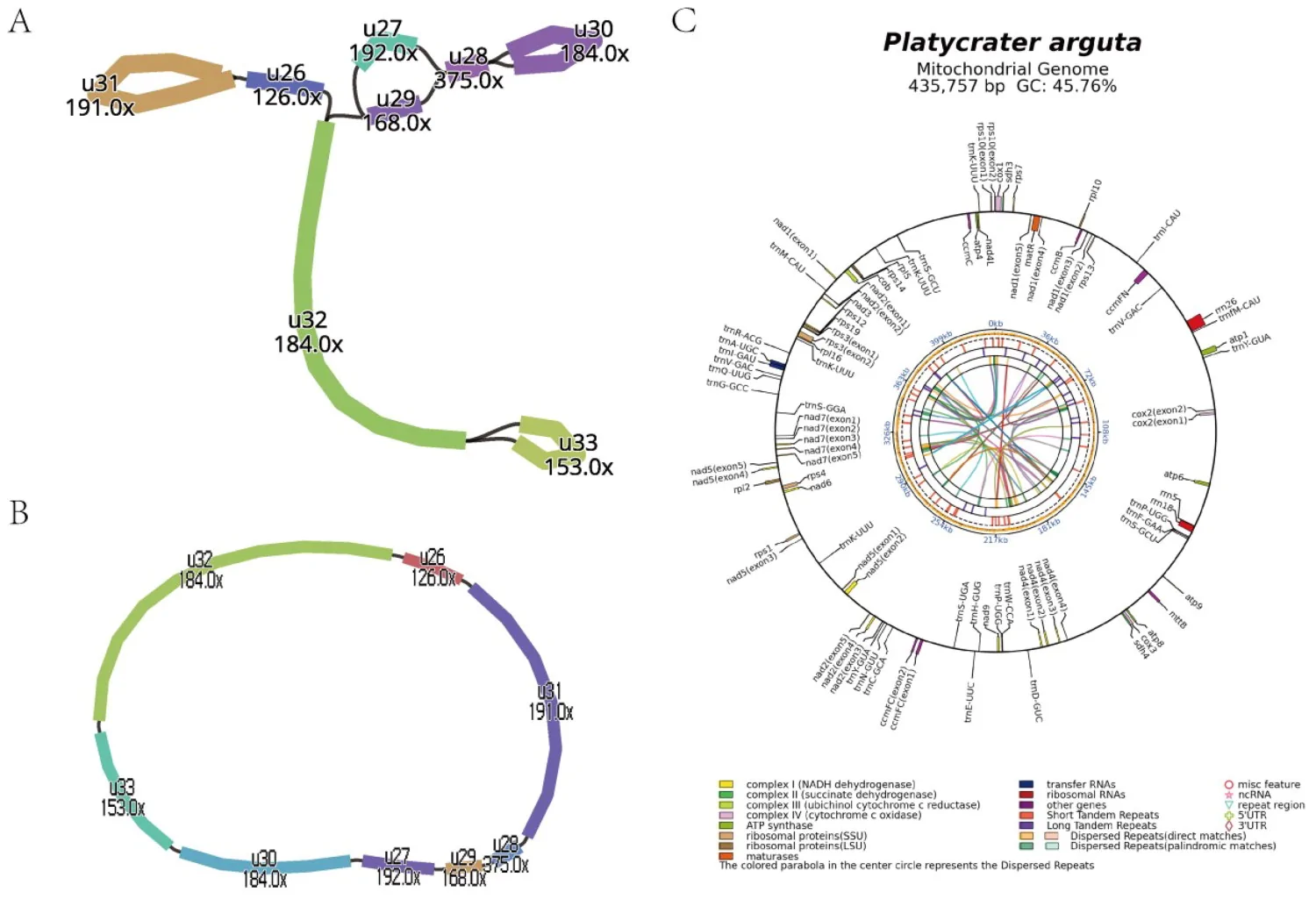
Structure and composition of the *P. arguta* mitogenome. (A) Initial assembly graph showing complex topology. (B) Resolved master circular conformation after contig ordering based on long-read evidence. (C) Final mitogenome map with genes color-coded by functional category; outer circle genes transcribed clockwise, inner circle counterclockwise. Innermost lines indicate dispersed repeats; gray circle shows GC content.

The complete mitogenome of *P. arguta* was assembled into a master circular conformation of 435,757 bp, with a GC content of 45.76% (Figure 2, Supplemental Figure S1). Mapping of the 10-Gb PacBio HiFi reads confirmed a robust and continuous average sequencing depth of 324.91× across the final assembly (Supplemental Figure S2). The sequence has been deposited in GenBank under accession number PV985015. Compared with the previously reported *P. arguta* mitogenome (Zhang et al. 2025), our assembly revealed substantial genomic rearrangements (Supplemental Table S2, Supplemental Figure S3). This genome encoded 66 genes, including 36 protein-coding genes (PCGs), 27 tRNA genes, and 3 rRNA genes, plus one *rpl16* pseudogene. Trans-splicing events were detected in several genes (e.g. nad1, nad2, nad5) (Supplemental Figure S4).

Codon usage analysis revealed a significant RSCU bias across all protein-coding genes (Supplemental Figure S5). Among 30 codons with RSCU > 1.0, 28 (93.3%) ended in A or U, indicating a pronounced AT bias. For instance, UCA (Serine) exhibited the highest RSCU value of 1.64. Additionally, various repetitive sequences were identified (Supplemental Figure S6), including 100 large dispersed repeats (>50 bp), 26 tandem repeats, and 136 simple sequence repeats (SSRs). Four chloroplast-derived regions (MTPTs) with spanning 25,060 bp were identified, 5.8% of the mitogenome (Supplemental Table S3).

**Figure 3.**
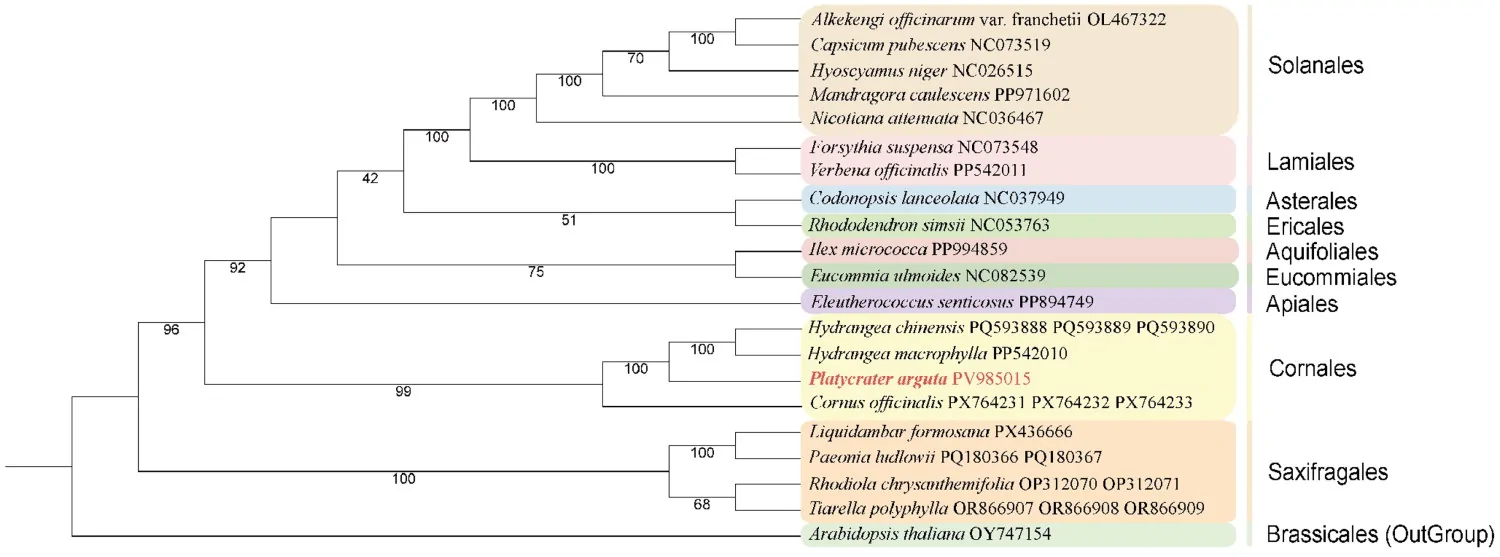
Phylogenetic tree based on shared protein-coding genes from 21 species, with *Arabidopsis thaliana* as the outgroup. Node numbers indicate bootstrap support values.The following sequences were used: *Alkekengi officinarum* var. franchetii (OL467322, unpublished), *capsicum pubescens* (NC073519, Li et al. 2024), *hyoscyamus Niger* (NC026515, Sanchez-Puerta et al. 2015), *mandragora caulescens* (PP971602, unpublished), *nicotiana attenuata* (NC036467, Xu et al. 2017), *forsythia suspensa* (NC073548, song et al. 2023), *verbena officinalis* (PP542011, unpublished), *codonopsis lanceolata* (NC037949, lee et al. 2018), *rhododendron simsii* (NC053763, Xu et al. 2021), *ilex micrococca* (PP994859, unpublished), *eucommia ulmoides* (NC082539, unpublished), *eleutherococcus senticosus* (PP894749, unpublished), *hydrangea chinensis* (PQ593888, PQ593889, PQ593890, Ye et al. 2025), *hydrangea macrophylla* (PP542010, unpublished), *platycrater arguta* (PV985015, This study), *cornus officinalis* (PX764231, PX764232, PX764233, olutayo et al. 2026), *liquidambar formosana* (PX436666, unpublished), *paeonia ludlowii* (PQ180366, PQ180367, zeng et al. 2025), *rhodiola chrysanthemifolia* (OP312070, OP312071, Yu et al. 2023), *tiarella polyphylla* (OR866907, OR866908, OR866909, Liu et al. 2024), and *Arabidopsis thaliana* (OY747154, unpublished). For species with multipartite mitogenomes (e.g. *H. chinensis* PQ593888–PQ593890, *C. officinalis* PX764231–PX764233, *T. polyphylla* OR866907–OR866909, *P. ludlowii* PQ180366–PQ180367, *R. chrysanthemifolia* OP312070–OP312071), shared protein-coding genes from all chromosomes were extracted and concatenated; each species is represented as a single terminal node, with all chromosome accessions listed.

To determine the evolutionary position of *P. arguta*, a maximum likelihood phylogenetic tree was constructed using protein-coding genes from 21 species. *P. arguta* was positioned within Hydrangeaceae, forming a sister group with *H. macrophylla* and *H. chinensis* with 100% bootstrap support (Figure 3).

## 4. Discussion and conclusion

In this study, we constructed the complete mitogenome of *P. arguta* (Hydrangeaceae) using PacBio HiFi sequencing and analyzed its structural features, gene content, repetitive sequences, and phylogenetic position.

The assembled mitogenome was a master circular molecule of 435,757 bp with a GC content of 45.76%, comparable to a previously reported *P. arguta* mitogenome from Wenzhou, Zhejiang (Zhang et al. 2025). However, the alternative, repeat-mediated subgenomic conformations in our study cannot be excluded. Horizontal comparison across published Hydrangeaceae mitogenomes highlighted highly plastic genome sizes (Supplemental Table S4). Our *P. arguta* mitogenome contained 66 functional genes, including 36 protein-coding genes, 27 tRNA genes, and 3 rRNA genes, essentially covering the core coding content of angiosperm mitochondria (Gualberto and Newton 2017). Trans-splicing events detected in several genes (e.g. *nad1*, *nad2*, *nad5*) indicated retention of complex splicing mechanisms of the *P. arguta* mitogenome, a significant feature of plant mitochondrial evolution. Whereas, these trans-splicing events would require additional structural or transcriptomic validation. Codon usage analysis of *P. arguta* mitochondria revealed distinct AT bias, with codons exhibiting RSCU > 1 predominantly ending in A or U, a pattern prevalent in other angiosperms (Sloan et al. 2012). Additionally, the 136 identified SSRs provide candidate molecular markers for future genetic-diversity, phylogeographical, or conservation studies.

The phylogenetic analysis placed *P. arguta* within Hydrangeaceae, forming a highly supported sister-group relationship with *H. macrophylla* and *H. chinensis*. This validates the systematic position of *P. arguta* and supports the efficacy of mitochondrial protein-coding genes for phylogenetic inference (Qiu et al. 2010; Yang et al. 2023). This study refines our understanding of the *P. arguta* mitogenome, providing a valuable genetic resource for comparative genomics and phylogenetic research. Future investigations may integrate nuclear and chloroplast genomic data to explore gene transfer patterns between organelles and their implications for functional genome structure.

## Supplementary Material

Supplemental_final.docx

## Data Availability

The complete mitogenome sequence reported in this article has been submitted to the GenBank database of NCBI under the accession number PV985015. The raw genomic sequencing data have been deposited in the Genome Sequence Archive (GSA) of the National Genomics Data Center (NGDC) under project accession number PRJCA044124 and run accession number CRA02872
