# Supplementary material for: Complete mitogenome and phylogenetic analysis of the tertiary relict shrub Platycrater arguta (Hydrangeaceae)
Source: Mitochondrial DNA B Resour. 2026 Sep 18;11(10):1187–91. doi: 10.1080/23802359.2026.2732717 (PMC13591951; doi:10.1080/23802359.2026.2732717)
Supplement: Supplementary file 1 [file TMDN_A_2732717_SM7716.docx]

**Supplemental Table S1**. Species used in this study and their corresponding GenBank accession numbers.

| **Order** | **Species** | **Accession No.** | **Source** |
| --- | --- | --- | --- |
| *Solanales* | *Alkekengi officinarum* var. franchetii | OL467322.1 | Unpublished |
| *Solanales* | *Capsicum pubescens* | NC_073519.1 | Li et al. 2024 |
| *Solanales* | *Hyoscyamus niger* | NC_026515.1 | Sanchez-Puerta et al. 2015 |
| *Solanales* | *Mandragora caulescens* | PP971602.1 | Unpublished |
| *Solanales* | *Nicotiana attenuata* | NC_036467.1 | Xu et al. 2017 |
| *Lamiales* | *Forsythia suspensa* | NC_073548.1 | Song et al. 2023 |
| *Lamiales* | *Verbena officinalis* | PP542011.1 | Unpublished |
| *Asterales* | *Codonopsis lanceolata* | NC_037949.1 | Lee et al. 2018 |
| *Ericales* | *Rhododendron simsii* | NC_053763.1 | Xu et al. 2021 |
| *Aquifoliales* | *Ilex micrococca* | PP994859.1 | Unpublished |
| *Eucommiales* | *Eucommia ulmoides* | NC_082539.1 | Unpublished |
| *Apiales* | *Eleutherococcus senticosus* | PP894749.1 | Unpublished |
| *Cornales* | *Hydrangea chinensis* | PQ593888.1, PQ593889.1, PQ593890.1 | Ye et al. 2025 |
| *Cornales* | *Hydrangea macrophylla* | PP542010.1 | Unpublished |
| *Cornales* | *Platycrater arguta* | PV985015.1 | This study |
| *Cornales* | *Cornus officinalis* | PX764231.1, PX764232.1, PX764233.1 | Olutayo et al. 2026 |
| *Saxifragales* | *Liquidambar formosana* | PX436666.1 | Unpublished |
| *Saxifragales* | *Paeonia ludlowii* | PQ180366.1, PQ180367.1 | Zeng et al. 2025 |
| *Saxifragales* | *Rhodiola chrysanthemifolia* | OP312070.1, OP312071.1 | Yu et al. 2023 |
| *Saxifragales* | *Tiarella polyphylla* | OR866907.1, OR866908.1, OR866909.1 | Liu et al. 2024 |
| *Brassicales* | *Arabidopsis thaliana* | OY747154.1 | Unpublished |

**References:**

Li, L., Fu, H., Altaf, M. A., Wang, Z., & Lu, X. (2024). The complete mitochondrial genome assembly of *Capsicum pubescens* reveals key evolutionary characteristics of mitochondrial genes of two Capsicum subspecies. *BMC Genomics*, 25(1), 1064. https://doi.org/10.1186/s12864-024-10985-w

Sanchez-Puerta, M. V., Zubko, M. K., & Palmer, J. D. (2015). Homologous recombination and retention of a single form of most genes shape the highly chimeric mitochondrial genome of a cybrid plant. *New Phytologist*, 206(1), 381–396. https://doi.org/10.1111/nph.13188

Xu, Y., Zhang, F., & Hu, K. (2017). The complete mitochondrial genome sequence of an annual wild tobacco *Nicotiana attenuata*. *Mitochondrial DNA Part B: Resources*, 2(2), 924–925. https://doi.org/10.1080/23802359.2017.1407946

Song, Y., Du, X., Li, A., Fan, A., He, L., Sun, Z., Niu, Y., & Qiao, Y. (2023). Assembly and analysis of the complete mitochondrial genome of *Forsythia suspensa* (Thunb.) Vahl. *BMC Genomics*, 24(1), 708. https://doi.org/10.1186/s12864-023-09821-4

Lee, H. O., Choi, J. W., Baek, J. H., Oh, J. H., Lee, S. C., & Kim, C. K. (2018). Assembly of the mitochondrial genome in the Campanulaceae family using Illumina low-coverage sequencing. *Genes*, 9(8), 383. https://doi.org/10.3390/genes9080383

Xu, J., Luo, H., Nie, S., Zhang, R. G., & Mao, J. F. (2021). The complete mitochondrial and plastid genomes of *Rhododendron simsii*, an important parent of widely cultivated azaleas. *Mitochondrial DNA Part B: Resources*, 6(3), 1197–1199. https://doi.org/10.1080/23802359.2021.1903352

Ye, K., Qin, J., & Yonghong, H. (2025). Decoding the complete mitochondrial genome of *Hydrangea chinensis* Maxim.: Insights into genomic recombination, gene transfer, and RNA editing dynamics. *BMC Plant Biology*, 25, 1078. https://doi.org/10.1186/s12870-025-07119-z

Olutayo, M. T., Jiang, Z., Zhou, H., Wang, H., & Zhang, H. (2026). The complete mitochondrial genome of *Cornus officinalis* reveals a multipartite structure and clarifies its phylogenetic position. *Mitochondrial DNA Part B: Resources*, 11(5), 659. https://doi.org/10.1080/23802359.2026.2658962

Zeng, Z., Zhang, Z., Norbu, N., Bonjor, N., Tan, X., Zhang, S., Tso, N., Wang, J., & Qiong, L. (2025). Comprehensive analysis of the complete mitochondrial genome of *Paeonia ludlowii* reveals a dual-circular structure and extensive inter-organellar gene transfer. *Biology*, 14(7), 854. https://doi.org/10.3390/biology14070854

Yu, X., Wei, P., Chen, Z., Li, X., Zhang, W., Yang, Y., Liu, C., Zhao, S., Li, X., & Liu, X. (2023). Comparative analysis of the organelle genomes of three *Rhodiola* species provide insights into their structural dynamics and sequence divergences. *BMC Plant Biology*, 23(1), 156. https://doi.org/10.1186/s12870-023-04159-1

Zhang, X., et al. (2024). Two chromosome-level genome assemblies of *Rhodiola* shed new light on genome evolution in rapid radiation and evolution of the biosynthetic pathway of salidroside. The Plant Journal, 117(2), 464–482.

Liu, B., Long, Q., Lyu, W., Shi, Y., Li, P., & Liu, L. (2024). Characterization of the complete mitogenome of *Tiarella polyphylla*, commonly known as Asian foamflower: Insights into the multi-chromosomes structure and DNA transfers. *BMC Genomics*, 25(1), 883. https://doi.org/10.1186/s12864-024-10790-5

**Supplemental Table S2.** Pairwise metric comparison between the PV985015 mitogenome (this study) and the Zhang 2025 *Platycrater arguta* record (two chromosomes).

| **Metric** | **PV985015 (this study)** | **Zhang 2025 (Feng & Zhang)** |
| --- | --- | --- |
| Sample source | Baishanzu Town, Qingyuan County, Lishui City, Zhejiang Province, China (27°45.08′N,119°11.56′E) | Ouhai District, Wenzhou, China (27◦99′ N, 120◦63′ E) |
| Sequencing platform | PacBio HiFi (coverage 324.91x, based on a 10 Gb subset; assembled with PMAT v2.1.5 + OATK v1.0) | PacBio (coverage 197.5x; assembled with PMAT2 v2.0.2 + Bandage v0.8.1) |
| Total length & conformation | 435,757 bp; one circular chromosome (single circular molecule) | 521,970 bp; two linear chromosomes (chr1 272,565 + chr2 249,405) |
| Protein-coding genes (CDS) | 36 | 35 |
| tRNA / rRNA | 27 / 3 | 27 / 3 |
| Total gene number | 132 | 131 |
| GC content | 45.76% | 45.62% |
| Repeat count (≥ 100 bp exact repeats) | 9 regions (3,509 bp, 0.81%) | 22 regions (29,444 bp, 5.64%) |
| Collinearity / rearrangements | The two assemblies are highly homologous but frequently rearranged; no single large collinear block exists between the single circular PV985015 and the two-chromosome Zhang assembly (largest block ~37 kb) | Same as left (the chr1/chr2 boundary at 272,565 bp is marked with a dashed line in the figure) |

**Supplemental Table S3. Chloroplast-derived regions (MTPTs) identified in the *Platycrater arguta* mitogenome.** MTPTs were identified by BLASTn (e-value ≤ 1e-5) using the *P. arguta* chloroplast genome (GenBank accession 2599487348) as query against the mitogenome; overlapping high-scoring pairs (HSPs) were merged into distinct regions, and regions >100 bp and >90% identity were retained as MTPTs.

| **Region** | **Chloroplast coordinates (bp)** | **Length (bp)** | **Identity (%)** | **Orientation** | **Mitogenome locus (bp)** |
| --- | --- | --- | --- | --- | --- |
| R1 | 31,668–31,793 | 126 | 93.7 | − | 207,657–207,782 (−) |
| R2 | 42,167–47,250 | 5,084 | 99.6 | − | 332,541–337,625 (−) |
| R3 | 102,028–111,452 | 9,425 | 99.9 | +/− | 57,858–60,377 (−); 343,592–352,823 (+/−) |
| R4 | 133,182–142,606 | 10,425 | 99.9 | +/− | 57,858–60,377 (+/−); 343,592–352,823 (−) |
| Total | — | 25,060 | — | — |  |

MTPTs total 25,060 bp, ca.5.8% of the 435,757 bp mitogenome (range 126–10,425 bp; identity 93.7–100%). R3 and R4 correspond to the two inverted-repeat (IR) copies of the chloroplast rRNA operon and share the same mitogenome insertion loci; orientation is relative to the chloroplast query, with +/− denoting mixed-strand mapping.

**Supplemental Table S4.** Cross-species comparison of five Hydrangeaceae mitochondrial genomes (genome size, gene content, repeat composition).

**Table S4A** Unified-method parsing (repeat ≥ 100 bp exact repeat regions, consistent with the PV985015 method in this study).

| **Species (record)** | **Source** | **Size (bp)** | **Conformation** | **GC%** | **CDS** | **tRNA** | **rRNA** | **Repeat regions (**≥ **100bp)** | **Total repeat length / %** |
| --- | --- | --- | --- | --- | --- | --- | --- | --- | --- |
| *P. arguta* (PV985015) - this study | This study | 435,757 | 1 circle | 45.76 | 36 | 27 | 3 | 10 | 3,311 bp (0.76%) |
| *P. arguta* (Zhang 2025) | Zhang 2025 | 521,970 | 2 linear | 45.62 | 37* | 27 | 3 | - | 79,506 bp (15.23%) |
| *H. macrophylla* (PP542010) | GenBank (unpublished) | 599,499 | 1 circle | 45.24 | 31 | 16 | 3 | 13 | 3,141 bp (0.52%) |
| *H. chinensis* (Ye 2025) | Ye 2025 / GenBank | 722,918 | circle + linear (3 chr) | 45.33 | 39 | 31 | 3 | 18 | 3,146 bp (0.44%) |
| *H. macrophylla* 'Endless Summer' (Wen 2026) | Wen 2026 / GenBank | 773,669 | 2 circles (2 chr) | 45.25 | 42 | 31 | 3 | 24 | 12,854 bp (1.66%) |

**Table S4B**. Key composition reported by each publication (different criteria; for text description only, not for direct cross-column comparison).

| **Species** | **Repeats reported in paper** | **RNA editing** | **MTPT** | **Note** |
| --- | --- | --- | --- | --- |
| *P. arguta* PV985015 | 100 dispersed (> 50bp) + 26 tandem + 136 SSR | - | see manuscript (~25.1 kb) | This study |
| *P. arguta* (Zhang 2025) | 79,506 bp (15.23%) | 687 sites | 31,177 bp | Zhang 2025 |
| *H. chinensis* (Ye 2025) | 335 repeat pairs + 211 SSR | 613 (C->U) | 23 regions / 11,059 bp | Ye 2025 |
| *H. macrophylla* 'Endless Summer' (Wen 2026) | 712 (227 SSR + 32 tandem + 453 dispersed) | 146 (26 PCGs) | 31 regions | Wen 2026 |
| *H. macrophylla* (PP542010) | not reported | not reported | not reported | GenBank record only |

| A   | B   |
| --- | --- |

**Supplemental Figure S1.** (A) The assembly graph of the *Platycrater arguta* mitogenome from de novo assembly.(B) Green segments represent single-copy regions; yellow highlights denote repeat elements; bidirectional arrows indicate regions capable of circularization; blue connecting lines illustrate the circular genome structure.

**Supplemental Figure S2.** Sequencing read coverage depth across the *Platycrater arguta* mitogenome. The y-axis represents the mean read depth, and the X-axis represents the genomic position (bp). The red dashed line indicates the overall mean depth of 324.91×.

**Supplemental Figure S3.** EasyFig (BLASTn) synteny comparison between PV985015 (this study) and the Zhang *et al.* 2025 *Platycrater arguta* record (two chromosomes), with chr1 (PV878598) on the top track, PV985015 (this study, the longest) on the middle track, and chr2 (PV878599) on the bottom track, showing homologous blocks and rearrangements.

**Supplemental Figure S4**. Structure of the trans-spliced genes *nad1*, *nad2*, and *nad5* in the mitogenome of *Platycrater arguta*. The figure illustrates the exons distributed across the genome and the mature mRNA produced after splicing.

**Supplemental Figure S5.** Heatmap of the Relative Synonymous Codon Usage (RSCU) for all protein-coding genes. Codons with RSCU values greater than 1 are shown in shades of red, while those with values less than 1 are shown in shades of blue. The color intensity corresponds to the RSCU value.

**Supplemental Figure S6.** Analysis of repeat sequences in the *Platycrater arguta* mitogenome.(A) Number and types of large dispersed repeats, including forward, palindromic, and tandem repeats, identified by REPuter. (B) Number and distribution of simple sequence repeats (SSRs) categorized by the repeat unit type, identified by MISA.
